# Soil-Dwelling Bacteria Display Tolerance Against Typical Antibiotic Serum Levels in an *Ex Vivo* Model of Traumatized Tissue

**DOI:** 10.1093/milmed/usaf340

**Published:** 2025-09-06

**Authors:** Annika L Gilmore, Lousili Peniata, Nicholas N Ashton, Dustin L Williams

**Affiliations:** Department of Biomedical Engineering, University of Utah, Salt Lake City, UT 84112, United States; Department of Orthopaedics, University of Utah, Salt Lake City, UT 84108, United States; Bone and Biofilm Research Laboratory, University of Utah, Salt Lake City, UT 84112, United States; Department of Orthopaedics, University of Utah, Salt Lake City, UT 84108, United States; Bone and Biofilm Research Laboratory, University of Utah, Salt Lake City, UT 84112, United States; Department of Orthopaedics, University of Utah, Salt Lake City, UT 84108, United States; Bone and Biofilm Research Laboratory, University of Utah, Salt Lake City, UT 84112, United States; Department of Biomedical Engineering, University of Utah, Salt Lake City, UT 84112, United States; Department of Orthopaedics, University of Utah, Salt Lake City, UT 84108, United States; Bone and Biofilm Research Laboratory, University of Utah, Salt Lake City, UT 84112, United States; Department of Pathology, University of Utah, Salt Lake City, UT 84112, United States; Department of Physical Medicine and Rehabilitation, Uniformed Services University of the Health Sciences, Bethesda, MD 20814, United States

## Abstract

**Introduction:**

Military wounds often contain large amounts of necrotic, avascular tissue that provide an ideal home for environmental and endogenous flora to dwell. Debridement mitigates infection but cannot be performed until the casualty is transported to a surgically equipped site, sometimes days after initial injury. Meanwhile, opportunistic pathogens spread throughout the wound site. Systemic antibiotic therapies fail to eradicate these soil microorganisms potentially because they dwell in an antibiotic-tolerant phenotype. Laboratory tests that incorporate environment soil may promote the development of point-of-care therapies effective against biofilm-like organisms. We hypothesized that, in an *ex vivo* model of traumatized tissue, blood-serum antibiotic concentrations would fail to eradicate soil-dwelling organisms.

**Materials and Methods:**

Sterile, excised tissue was collected from concurrent sheep studies. Tissue samples were coated with topsoil that was confirmed to contain naturally occurring viable organisms, then blasted with an air cannon to simulate trauma and distribute soil throughout. The tissue test environment was then moved into a 6 well-plate for treatment with saline suspensions of systemic levels of antimicrobials, or a 10× concentration of the same. Following a 24-hour incubation, tissues and remaining treatment was diluted and quantified on selective and non-selective agar plates. Colony forming units (CFU)/g of tissue were calculated between agar type and treatment group than compared using a Student’s *t*-test.

**Results:**

Non-treated, soil-contaminated tissues had an average of 8.43 ± 0.36 log_10_ CFU/g tissue. Fungal species were the most common organism cultured among all groups, though this may change between soil samples. Serum-level antimicrobials failed to reduce bioburden to a significant degree. Only 10× systemic concentrations achieved statically significant (*P *< .001) bioburden reduction and lowered organisms to below 5 log_10_ CFU/g tissue.

**Conclusions:**

Outcomes suggested that antimicrobial levels that would typically be administered prophylactically fail to kill soil microbes dwelling in compromised tissue. The findings support the premise for local, high-dose antimicrobial therapies capable of managing biofilm-life bacterial contamination. These results translate towards improved triage methods: to prolong the time from injury to debridement, anti-infective technologies must have the potential to kill organisms that otherwise tolerate low levels of antimicrobials. Systemic prophylaxis alone may be insufficient.

## INTRODUCTION

Soil is home to a diversity of microorganisms that aid agriculture, mitigate disease, and cycle nutrients. However, when soil microbes are dislodged from their natural environment and placed into an open wound, opportunistic pathogenicity may result. Injuries sustained in austere environments, such as the battlefield, are often highly contaminated with environmental and endogenous debris, contributing to heightened infection rates.^1^^,^^2^ Further, ballistic trauma can cause bone fractures and vasculature damage, hindering the patient’s immune response and creating necrotic surfaces ideal for bacterial colonization.^3^^,^^4^ Military personnel go to great lengths to reduce triage time, thoroughly debride wounds, and implement antimicrobial therapies, yet infection remains a challenging and expensive complication post-resuscitation.^5^ This persistent problem may be related to wound site contamination by soil-dwelling organisms and insufficient antibiotic serum levels to kill bacteria dwelling in an antibiotic-tolerant phenotype.

Soil microbial composition changes geographically and seasonally, as evidenced by wound cultures taken during conflict. *Staphylococcus* and *Streptococcus* organisms were present year-round in 30-60% of cases sustained during the Korean and Vietnam Wars, whereas Gram-negative organisms like *Escherichia coli, Bacillus*, *Enterobacter*, and *Pseudomonas* became more prevalent in wounds sustained during the summer or fall.^6^^,^^7^ Among casualties during Operations Iraqi Freedom and Enduring Freedom, invasive fungal infections resulted almost exclusively from injuries sustained in the Southern agricultural region.^8^ Similar to Vietnam, wounds of US military personnel treated at Landstuhl Medical harbored Gram-positive organisms year-round, but had an increase in Gram-negative, anaerobic, and multidrug-resistant Gram-negative pathogens during the summer and fall.^9^ Often, swabbed isolates are used to screen antibiotic resistance. Though minimal work has been undertaken to explore phenotypic tolerance of environmental pathogens to antibiotic treatment.

As conflicts become more hostile and remote, the time from injury to debridement may increase. Alternative methods are required to microbiologically stabilize wound sites during longer triage times. Models that incorporate environmental and endogenous flora can replicate future infection risks and allude to the biofilm-forming potential of microorganisms. Such information may encourage development and deployment of anti-­infective technologies.

Importantly, bacteria in natural ecosystems, such as soil, preferentially dwell in a biofilm phenotype.^10^^,^^11^ Bacterial biofilms exhibit antibiotic tolerance and are notoriously difficult to kill, often requiring 1,000× more antibiotics to have an effect compared to planktonic cells.^12–15^ Such characteristics may reflect production of extracellular polymeric substances, excreted defense enzymes, and altered metabolic states.^15^^,^^16^ These defenses protect aggregated bacteria from immune response and antibiotic attack and prevent traditional antibiotic regimens from fully eradicating bacteria.

Current prophylactic therapies are limited in their ability to kill biofilm or bioaggregates as these are routinely dosed at systemic concentrations to avoid toxic side effects. Communities of bacteria are phenotypically tolerant to clinical antibiotic regimes, despite having susceptibility to the same treatment when tested *in vitro* as planktonic isolates.^17^ Further, prophylactic antibiotics may not reach a wound site because of tissue ischemia. Elevated antibiotic concentrations are likely required to manage biofilm-related infection but are not currently used prophylactically for civilian trauma or combat casualty care.

In civilian sectors, the American College of Surgeons recommends cefazolin for prophylactic use in Gustilo fracture grades I and II. An adjunct aminoglycoside, such as gentamicin, is often added for grade III fractures.^18^ Military service members are more likely to receive either oral moxifloxacin or intravenous ertapenem, per the Tactical Combat Casualty Care guidelines.^19^ Nevertheless, in civilian and military severe open fracture injuries, early antibiotic administration is recommended,^20^ and a race to the operating room ensues. Although a “Golden Hour” evacuation time exists, with the goal of relocating injured soldiers to triage in a timely manner, there are often delays in adequate orthopedic treatment.^21^ This is expected to worsen in future conflicts.^22^ Biofilm-targeting therapies are required to microbiologically stabilize wound sites during multi-day transport to an equipped operating room. Such advancement, however, is dependent on the creation of relevant biofilm-related infection models.

Although numerous methods exist to grow laboratory biofilm,^23–25^ most produce monomicrobial biofilm and use inert growth substrates such as glass or polycarbonate to screen antibiotic therapies. Environmental biofilms, such as those dwelling within dirt or skin, are complicated by unique microenvironments, nutrient gradients, foreign bodies, and polymicrobial interactions that are difficult to produce and study in lab.^26–28^ Use of environmental soil and damaged tissue samples may better replicate real-world scenarios for the development and comparison of biofilm-targeting therapies by providing a diversity of organisms. In this study, we evaluated the infectious potential of topsoil-dwelling bacteria in an excised tissue model representative of traumatized soft tissue. We hypothesized that in an *ex vivo* model of traumatized tissue, soil-­dwelling microbes would display phenotypic tolerance to antibiotic concentrations commonly reached in serum.

## METHODS

### Topsoil and Excised Tissue Preparation

**Figure 1.**
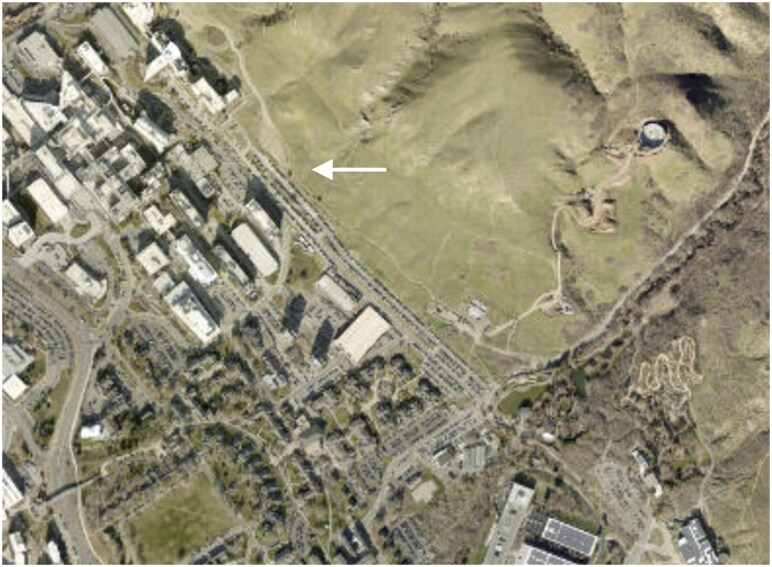
Aerial view of the University of Utah campus with a white arrow pointing to the location of soil collection. Minimally disturbed topsoil was used to contaminate excised tissue. Satellite image of the University of Utah campus where foothills of the Wasatch Mountains can be seen in the upper right. Soil was collected from a hiking trail in a location indicated by the white arrow. This location yielded silty soil.

Topsoil was sourced 12 hours before use from the foothills of the Wasatch Range near the University of Utah (**Figure 1**) and sieved through mesh with 2 mm openings to remove large particles.

Fresh ovine muscle tissue was obtained from concurrent protocols approved by the University of Utah’s Institutional Animal Care and Use Committee. The right hind limb was disarticulated then cooled in a commercial fridge for 1-3 hours before dissection to increase muscle stiffness. Wool was clipped and the remaining anterior skin was prepared with 3× alternating applications of 10% povidone-iodine solution and 70% isopropyl alcohol. To reduce endogenous contamination, 2 sets of surgical tools were used. First, a scalpel and forceps were used to remove skin, exposing muscle and fascia. Following skin removal, the second tool set was used to collect thirty muscle sections, approximately 1 × 1 × 0.5 cm^3^. Each tissue was oriented with vertical cross-sections facing upwards. Sections were placed onto sterile stainless-steel trays for soil contamination and blasting.

**Figure 2.**
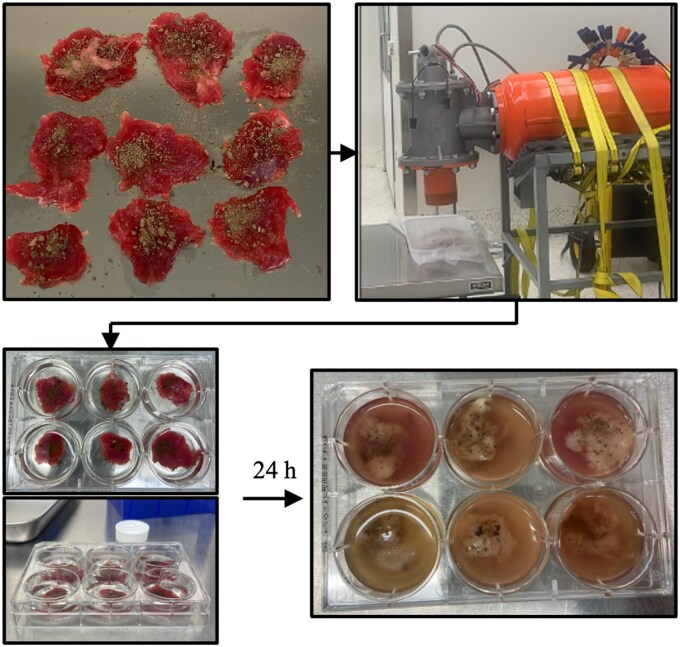
Flow chart description of methodology from *ex vivo* contamination to blast and quantification. Tissue sections weighing 3-5 g were dissected and topped with 200 µL of topsoil. The tissues were covered to create an enclosed, microbiologically protected environment. A Martin Air Cannon was used to blast soil into tissue samples to mimic ballistic trauma and evenly distribute debris and microbes throughout the test environment. Tissues were treated in 6 mL of phosphate buffered saline in individual 6-well plates. The entire system (e.g., tissue and treatment liquid) was quantified 24 hours later using a method to mitigate residual antimicrobial kill.

A volume of 200 µL of sieved soil was evenly spread across the top of each tissue. Trays containing 9 soil-contaminated tissues each were covered with sterile aluminum foil and blasted with a Martin Tornado air cannon charged to 30 psi.^29^ Thirty psi was the pressure determined experimentally to adequality push debris into tissues without tearing or compromising the aluminum cover or tearing apart excised tissue pieces (**Figure 2**). Covering was essential to limit microbiological contamination to collected soil samples, not debris particles from the cannon or surrounding environment.

Three process control tissues were quantified throughout the study (**Table 1**). The first was quantified immediately following dissection to determine if unwanted contamination occurred. The second was quantified after 24 hours incubation at 37 °C, and the third after a blast and 24 hours incubation at 37 °C. Samples were stomached (Seward; West Sussex, United Kingdom) at 4k for 3 minutes, the resulting solution was diluted and plated onto Columbia blood agar (Hardy Diagnostics; CA, United States).

**Table 1.** Elements of Each Test Groups, Including Antimicrobial Concentrations, and Process Controls Taken Throughout the Procedure

Group 1: Non-treated control	Group 2: Clinical treatment	Group 3: 10× treatment	Process controls
Blasted tissue + soil	Blasted tissue + soil	Blasted tissue + soil	1. NX tissue 2. Post-NX tissue 3. Post-blast tissue
PBS	60 µg/mL C 10 µg/mL G 30 µg/mL AB	600 µg/mL C 100 µg/mL G 300 µg/mL AB	NA

A condensed description of 3 study arms including the materials quantified from each and the corresponding antimicrobials and concentrations used to treat each group. Cefazolin (C), gentamicin (G), and amphotericin B (AB) were used to treat both Groups 2 and 3, though in different concentrations. Primary antibiotic suspensions were made in sterile water with further dilutions and final suspensions occurring in phosphate buffered saline (PBS). Process controls were taken throughout necropsy (NX) and before and after blast to screen for unintentional contamination.

### Antibiotic Cocktail Preparation

The concentrations of antimicrobial in the cocktail were based on average blood serum levels that might be in a typical prophylaxis scenario, that is the level of antimicrobials that contaminating organisms in soil might be exposed to if they resided in a traumatic wound site.^30–32^ This model did not necessarily account for damaged vasculature that might further disrupt antibiotic transport; the literature cited and concentrations selected reflect a “best case scenario” for systemic antibiotic administration and reflect peak blood serum concentration. We recognized that this cocktail, particularly with the addition of amphotericin B, would not necessarily be used clinically, but it represented common prophylactics used to mitigate infection risk, and the antifungal controlled fungal overgrowth in the experiment.

The systemic antibiotic cocktail (Group 2: Clinical treatment) included 60 µg/mL cefazolin, 10 µg/mL gentamicin, and 30 µg/mL liposomal amphotericin B (**Table 1**). Primary suspensions occurred in 20 mL of sterile water, then were further diluted in phosphate buffered saline (PBS) within a 50 mL tube.

A secondary cocktail (Group 3: 10× treatment) with 10× the concentrations listed above was also prepared (**Table 1**). The rationale was that the soil samples likely contained bioaggregates or biofilm-dwelling bacteria, which are known to display antimicrobial tolerance.^12^^,^^15^ The 10× cocktail more closely replicated local therapeutics that might be used in a trauma site, such as a local wound gel.

### Model Setup

Three study arms were established along with process controls (**Table 1**). The first test arm served as a positive control of growth. Aforementioned Group 1 soil-contaminated tissues were blasted and placed into a well of a 6-well plate with 6 mL PBS per well. Group 2 samples were likewise placed into a well of a 6-well plate with 6 mL PBS that contained the lower concentration cocktail. Group 3 samples were treated the same and exposed to the 10× cocktail. Tissues were placed overnight in a shaking incubator set to 37 °C and 40 rotations per minute. Each experiment was performed with *n* = 6 repeats.

### Bacterial Quantification

Following 24 hours incubation, tissues and solution were removed from a well and placed into a stomacher bag. Samples were homogenized at 400 rpm for 3 minutes. To account for residual antibiotic kill, we used a “spin down” quantification method; 1 mL of homogenized solution was added to a microcentrifuge tube and centrifuged at 12k rpm for 3 minutes. Then, 900 µL of supernatant was removed and replaced with sterile PBS. The solution was vortexed for 1 minute and the process was repeated twice more until sub-inhibitory concentrations of antibiotic remained.

Following spin down, samples were vortex-sonicated-vortexed for 1 minute, 10 minutes, and 10 seconds, respectively. Each sample was diluted 10-fold and plated onto Cetrimide (Neogen co.; MI, United States) to select for *Pseudomonas*, Baird Parker (Neogen co.; MI, United States) for *Staphylococcus*, Sauboraund (MP Biomedical; CA, United States) for fungal organisms and non-selective tryptic soy (TSA) (Sigma Aldrich; MO, United States) and Columbia blood agar plates for a comprehensive culture of soil-dwelling microbes. Plates were incubated at 37 °C overnight and counted the following day.

### Minimum Inhibitory Concentration Assay

To confirm that surviving organisms were susceptible to the antibiotic treatment, surviving *Staphylococcus* and *Pseudomonas* colonies were further isolated on TSA plates using a standard microbiological streak plate dilution. Isolated colonies were then screened for gentamicin and cefazolin susceptibility using an Minimum Inhibitory Concentration (MIC) assay. This was an important step to determine if surviving colonies were genotypically resistant to treatment or phenotypically tolerant.

Minimum Inhibitory Concentration were performed as described in Clinical & Laboratory Standards Institute Protocol M100. Briefly, 50 µL of Cation-Adjusted Mueller Hinton Broth were added to columns 2-12 of a 96-well plate and 50 µL of 256 μg/mL antibiotic in columns 1-2. A multichannel pipette was used to mix column 2 and add 50 µL of it to column 3 for a 1:2 dilution. Mixing continued until column 11. The final antibiotic concentration ranged from 64 to 0.0125 μg/mL. Fifty µL of 0.5 McFarland standard was added to each well in columns 2-12. The plate was covered in a plastic bag and incubated overnight at 37 °C. MICs were defined as the column and concentration in which no bacterial growth was present.

### Statistical Methods

Colony counts from selective and non-selective agar plates were log_10_ transformed and calculated as Colony forming units (CFU)/g of sample. CFU/g results of samples from the same group and agar-type were averaged, for a total of 15 bioburden averages. A Shapiro-Wilk test was used to identify data normality. Normally distributed data (all groups quantified on non-­selective agar) was assessed with a Student’s *t*-test (alpha = 0.05). Data lacking normality (Groups 2 and 3 Baird-Parker and Cetrimide) was compared with a Wilcoxon rank sum test.

## RESULTS

### The Model

No growth was detectable in any process control. This suggested that using separate tools to remove skin and collect tissue samples allowed us to successfully excise and blast tissues without compromising sterility.

Placement of the leg into a fridge before necropsy increased the stiffness of muscles and aided tissue sample collection. Without this step, the muscle was difficult to consistently trim. Some size variance remained, as samples weighed between 3 and 4 g. Tissues did noticeably degrade during incubation; they had a red, healthy appearance initially, but turned brown and partially disintegrated after 24 hours when contaminated with soil.

### Bacterial Quantification

**Figure 3.**
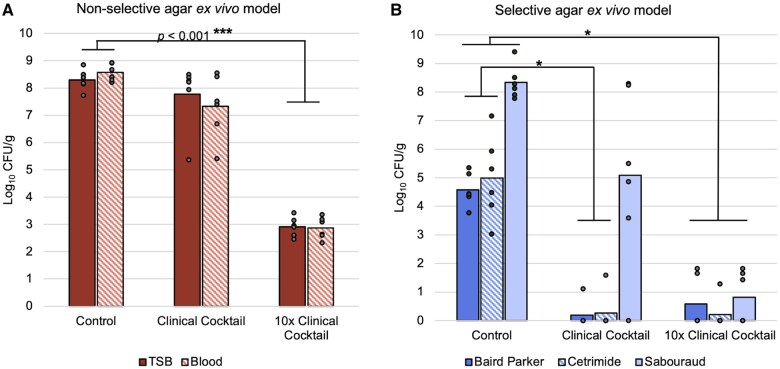
Average Colony forming units of each group (*n* = 6 per group) on non-selective (Trypic Soy Broth (TSB) and Columbia blood agar (Blood)) and selective (Sabourand, Cetrimide, Baird Parker) agar plates. (A) Solid bars represent average log_10_ CFU/g of tissue quantified from TSB plates; striped bars are the average from blood agar. An average of 8.43 ± 0.36 log_10_ CFUs/g of tissue were detected from non-selective agar plates. There was minimal Colony forming units reduction when samples were treated with a clinically relevant antimicrobial cocktail but a significant reduction (*P* < .001) of 5 log_10_ CFU when treated with 10× antimicrobials. (B) Leftmost solid dark blue bars represent the average log_10_ CFU/g of tissue of organisms isolated on Baird Parker, which is selective for staphylococcal organisms; striped blue bars from Cetrimide, selective for *Pseudomonas*; and rightmost solid light blue bars from Sabouraud dextrose agar, selective for fungal species. Of organisms counted from selective agar plates, fungal species were most common across all groups. There was a significant reduction in *Staphylococcus* and *Pseudomonas* organisms when treated with the clinically relevant dose, and near complete reduction in organisms from all selective agar plates when 10× cocktail was used. Abbreviation: CFUs/g, colony forming units per gram.

Multiple isolates grew on Group 1 and 2 plates but appeared to be minimized by Group 3 treatment (**Figure 3**). Group 1, non-treated controls had an average of 8.43 ± 0.36 log_10_ CFU/g tissue when plated on non-selective agar. Fungal organisms were prevalent in Group 1 control tissues with an average of 8.34 ± 0.59 log_10_ CFU/g on sabouraud plates. Staphylococcal and pseudomonal species on respective selective agar had an average of 4.58 ± 0.57 log_10_ CFU/g tissue and 5.00 ± 1.46 log_10_ CFU/g tissue, respectively.

Group 2 tissues exposed to the lower cocktail concentration had an average of 7.55 ± 1.50 log_10_ CFU/g tissue on non-selective plates, indicating an average bioburden reduction of 0.78 log_10_ CFU/g compared to Group 1 controls, which was insignificant (*P *= .33). However, *Staphylococcus* and *Pseudomonas* in Group 2 samples were significantly reduced compared to Group 1 (*P *< .001). On 1 of 6 selective agar plates, 1.12 log_10_ CFU/g tissue of *Staphylococcus* and 1.59 CFU/g tissue of *Pseudomonas* remained, though no growth occurred on the 5 other selective Baird Parker or ­Cetrimide plates quantified from Group 2.

Bioburden reduction was notable in Group 3. Specifically, Group 3 samples had an average remaining bioburden of 2.89 ± 0.36 log_10_ CFU/g tissue on non-selective agar, which was greater than a 5 log_10_ reduction compared to Group 1 controls (*P *< .001). Only 2 out of 6 selective Baird Parker agar plates had *Staphylococcus* growth (1.66 and 1.83 log_10_ CFU/g). And only one selective cetrimide agar plate had *Pseudomonas* growth (1.28 log_10_ CFU/g). An average of 0.81 ± 0.90 log_10_ CFU/g fungal organisms were counted on selective Sabouraud. Overall, organisms, particularly *Staphylococcus*, *Pseudomonas*, and fungal, were significantly reduced by the 10× cocktail (*P* < .001). Interestingly, *ex vivo* tissue samples in Group 3 treatments appeared pinker and more intact than those of Groups 1 or 2.

### Minimum Inhibitory Concentration Assay

All but 2 isolated *Staphylococcus* organisms were susceptible to cefazolin, and all *Staphylococcus* and *Pseudomonas* isolates were susceptible to gentamicin (**Table 2**), suggesting phenotypic antibiotic tolerance rather than isolate resistance. Increased antibiotic doses were effective at significantly reducing bioburden in Group 3 alone, yet common isolates from each group were susceptible to treatment when tested in a planktonic phenotype.

**Table 2.** Minimum Inhibitory Concentration (MIC) of Isolated *Staphylococcus* and *Pseudomonas* Organisms From Each Group

Isolate	Cefazolin MIC (µg/mL)	Gentamicin MIC (µg/mL)
Group 1 *Staphylococcus*	0.5	2
Group 1 *Pseudomonas*	–	1
Group 2 *Staphylococcus*	>32	1
Group 2 *Pseudomonas*	–	1
Group 3 *Staphylococcus*	>32	1
Group 3 *Pseudomonas*	–	1

The MIC of cefazolin and gentamicin was determined against the most prevalent *Staphylococcus* organisms initially isolated from Baird Parker selective agar plates and *Pseudomonas* isolated from Cetrimide selective agar plates. All screened isolated were susceptible to gentamicin. This result suggest planktonic isolates are susceptible to antibiotic treatment, but communities of the same organisms display phenotypic tolerance. Cefazolin is not active against *Pseudomonas* and thus the MIC of these isolates was not tested. One *Staphylococcus* isolate was suspectable to cefazolin, though others were not. A broad-spectrum antibiotic may be warranted to better manage contaminating Gram-negative organisms.

## DISCUSSION

Time from injury to debridement is likely to increase in future conflicts.^22^ Elevated antibiotic concentrations applied locally may become common practice if triage delays occur. Before debridement, systemic antibiotics cannot reach the wound site, and devitalized tissue can quickly become host to contaminating environmental and endogenous flora. To develop and test technologies that may prolong the “golden hour,” models that use environmental sources could better replicate the relative infective risk and provide an intermediate screening method between *in vitro* and *in vivo* tests. Results from this study suggest soil microorganisms behave like biofilm by exhibiting tolerance to systemic antibiotic concentrations. Early microbiological management is required before hospital care and our results support higher antimicrobial concentrations as a potential management method.

Clinically, local antibiotic use is disputed as certain products or antibiotic doses can be toxic. Yet bacteria and infection are also problems. Thus, the cost to benefit ratio of local antibiotic application versus bioburden reduction must be weighed. Selection of appropriate safe and effective antibiotics for topical and parenteral use in open wound sites is recommended. Generally, the damage that bacterial biofilms can cause encourages exploration of local antibiotic delivery mechanisms for increased efficacy with as limited a toxic effect as possible.^14^^,^^15^^,^^18^

### The Model

The primary benefit of this study is the use of an *ex vivo* test environment. Tissue can create complex areas for bacteria to adhere and resist antibiotic threats resulting in a more challenging model than *in vitro* assays. However, obtaining sterile tissue can be troublesome. Replacement of tools throughout the collection procedure mitigated contamination as confirmed by process controls.

When larger tissues with non-standardized orientations were used, the results were skewed (data not shown) likely because of microscopic changes such as oxygen and nutrient gradients within the test environment.^33^ The orientation of tissues (e.g., all muscle cross sections oriented vertically), and increased control over tissue geometry while excising promoted consistency of bacterial quantification results. Maintaining the same orientation, and height, promoted even blast dispersal of the soil throughout the tissue, and reduced variation in bioburden between samples of the same group.

### Bacterial Quantification

Soil microbes blasted into excised tissue and exposed to a clinically relevant antimicrobial solution behaved phenotypically like biofilm by exhibiting antibiotic tolerance. Bioburden of the same soil organisms was reduced when exposed to elevated antibiotic concentrations. Localized antibiotic therapies may promote microbiological management of wound sites by increasing wound site antibiotic concentration without promoting systemic cytotoxicity.

We intentionally cultured and quantified organisms aerobically to replicate damaged tissue lacking a protective skin barrier that is often environmentally exposed. Combat wound cultures taken immediately after injury have some anaerobic bacteria, but often these organisms decrease in the following days.^34^ Although interventions such as debridement, irrigation, and antibiotic therapy may alter the composition of pathogens dwelling within a wound site, traumatic blast injuries often have high amounts of exposed surface area. Aerobic culture methods were fitting as informed by common infectious organisms documented in the literature.

Most colonies appeared to be either *Bacillus* or fungal organisms. This finding corroborates previous swab cultures,^6^^,^^9^^,^^35^ as the soil sample was collected during summer, following rainfall, in relatively “green” area. Had a sample been taken later in winter or early spring, the microbial type and density might have altered.

### Minimum Inhibitory Concentration Assay

The MIC susceptibility outcomes in combination with the disparity in CFU counts between the 1× and 10× antimicrobial cocktail support the likelihood that soil microorganisms resided in an antibiotic-tolerant biofilm-like phenotype. All screened isolates were suspectable to gentamicin, though non-*Staphylococcus* or *Pseudomonas* species could have exhibited resistance (not all organisms were isolated and tested). Future work should further parse microbial species and resistance profiles.

### Limitations and Future Work

A limitation of this approach is the lack of inoculum from endogenous skin flora. Further studies may incorporate *Staphylococcus aureus* or *Staphylococcus epidermidis* biofilm or planktonic susceptions to replicate endogenous wound site contamination. Inclusion of these organisms may alter bacterial recovery because of complex interactions between isolates. Incorporation of common skin microbes could offer a comprehensive understanding of bacterial wound site contamination. Similarly, future incorporation of plasma, rather than PBS, may better replicate traumatic injury and further complicate bacterial growth and antibiotic activity.

Amphotericin B was useful in this model to manage fungal growth in an attempt to promote other non-fungal organisms that might be more representative of battlefield wounds. However, higher concentrations could be used in future work as our results indicated an increased presence of fungal organisms compared to staphylococcal or *Pseudomonas* species. Further consideration of fungal treatments may be warranted as future point-of-injury products are developed and if conflicts occur in vegetative regions.

We did not genetically identify organisms but rather relied on MIC and growth characteristics to support our hypothesis that communal biofilm-like soil microbes tolerate antibiotic treatment. Genotyping organisms could reveal the prevenance of biofilm-forming genes and specific organisms could be more readily identified. The use of additional selective agars could also be used to determine the type and distribution of surviving organisms.

## CONCLUSIONS

We, and other biofilm researchers, routinely reference literature that suggests soil-dwelling microbes may be tolerant to serum levels of antibiotics, though minimal work has been undertaken to explore this hypothesis. Data from this study showed soil-dwelling microbes persist in excised tissue when exposed to clinically relevant antimicrobial concentrations, suggesting that elevated concentrations may be required to microbiologically manage soil-contaminated wound sites. This study promotes an understanding of how soil dwelling organisms persist in the presence of clinically relevant antibiotics and aids a broader awareness of the infection risk associated with contaminated wounds. Additionally, this model offers a realistic and microbiologically challenging bench-top method to screen anti-infective technologies and could be used to test local antibiotic ointments or sprays against realistic environmental contaminants.

## Data Availability

The data underlying this article will be shared on reasonable request to the corresponding author.

## INSTITUTIONAL ANIMAL CARE AND USE COMMITTEE

Tissue sharing from a concurrent Institutional Animal Care And Use Committee-approved study was permitted by the University of Utah Office of Comparative Medicine.
